# Differences in Perceived Occupational Stress by Demographic Characteristics, of European Emergency Medical Services Personnel during the COVID-19 Virus Pandemic—An International Study

**DOI:** 10.3390/healthcare9111582

**Published:** 2021-11-19

**Authors:** Tomasz Ilczak, Małgorzata Rak, Kacper Sumera, Carl Robert Christiansen, Esther Navarro-Illana, Pasi Alanen, Juha Jormakka, Elena Gurková, Darja Jarošová, Danka Boguská, Michał Ćwiertnia, Monika Mikulska, Wioletta Pollok-Wakmańska, Rafał Bobiński, Marek Kawecki

**Affiliations:** 1 Department of Emergency Medicine, Faculty of Health Sciences, University of Bielsko-Biała, Willowa 2, 43-309 Bielsko-Biała, Poland; malgorzatarak999@gmail.com (M.R.); mcwiertnia@ath.bielsko.pl (M.Ć.); mmikulska@ath.bielsko.pl (M.M.); mkawecki@ath.bielsko.pl (M.K.); 2 Faculty of Health Social Care & Medicine, School of Nursing Midwifery & Allied Health, Edge Hill University, Manchester M1 6FQ, UK; Kacper.Sumera@edgehill.ac.uk; 3 Faculty of Health Sciences, Oslo Metropolitan University, 0167 Oslo, Norway; carlro@oslomet.no; 4 Department of Nursing, Catholic University of Valencia, 46003 Valencia, Spain; esther.navarro@ucv.es; 5 Faculty of Social and Health Care, Seinäjoki University of Applied Sciences, 60100 Seinäjoki, Finland; pasi.alanen@lab.fi; 6 EMA Finland, Nuijamiestentie 3C, 00400 Helsinki, Finland; juha.jormakka@ema.fi; 7 Department of Nursing, Faculty of Health Sciences, Palacký University in Olomouc, CZ-77900 Olomouc, Czech Republic; elena.gurkova@upol.cz; 8 Department of Nursing and Midwifery, Faculty of Medicine, University of Ostrava, CZ-70300 Ostrava, Czech Republic; darja.jarosova@osu.cz; 9 Department of Urgent Health Care, Faculty of Health Care, University of Prešov in Prešov, SK-08001 Prešov, Slovakia; danka.boguska@unipo.sk; 10 Department of Public Health, Faculty of Health Sciences, University of Bielsko-Biała, 43-309 Bielsko-Biała, Poland; wwaksmanska@ath.bielsko.pl; 11 Department of Biochemistry and Molecular Biology, Faculty of Health Sciences, University of Bielsko-Biała, 43-309 Bielsko-Biała, Poland; rbobinski@ath.bielsko.pl

**Keywords:** occupational stress, emergency medical services, medical professionals, emergency procedures, COVID-19 pandemic, predictors of stress

## Abstract

Objectives: The outbreak of the COVID-19 pandemic has brought commercial, social, and economic consequences in every country that has experienced substantial SARS-CoV-2 infection rates. The complete change in the environment that took place due to the outbreak of the pandemic can lead to stressful situations, especially among healthcare personnel. Material and methods: The research were conducted during the COVID-19 pandemic between the 27 March 2020 and the 20 April 2020. The research included 1984 employees of emergency medical systems in seven European countries. An internet-based questionnaire format was adopted for the study (ΩMc-Donald > 0.7). Results: The highest level of stress was experienced by personnel in the United Kingdom M = 4.03, and the lowest by Norwegian employees M = 2.89. High levels of stress were also experienced by nurses from Spain and Poland. Women actively working in the healthcare system during the pandemic experienced higher stress levels than men. Conclusions: Women working in European emergency medical systems are more vulnerable to work-related stress, while carrying out emergency medical procedures during the pandemic. Differences in the level of stress experienced while carrying out duties in pre-hospital conditions were only found among Spanish emergency medical system personnel.

## 1. Introduction

The outbreak of the COVID-19 pandemic has brought commercial, social, and economic consequences in every country that has experienced substantial SARS-CoV-2 infection rates. One aspect of the pandemic that is often ignored for individual countries is its effect on the functioning of the healthcare system, which was faced with the task of conducting medical procedures in entirely new conditions. The complete change in the environment that took place due to the outbreak of the pandemic can lead to stressful situations, especially among healthcare personnel [1]. The WHO definition of work-related stress is the loss of control over situations related to carrying out professional duties [2]. An additional element that can generate stress is merely the fact of carrying out a medical profession in conditions of particular danger to one’s health or life. Performing emergency procedures under conditions of chronic stress may result in an increased number of errors and have health consequences for patients. As previous research has shown, healthcare personnel are particularly prone to work-related stress, and the most at-risk professional group are those “on the front line”, that is emergency medical response personnel [3,4]. Work-related stress can be exacerbated due to continuous exposure to infection by the virus through contact with infected people, additional work duties related to the increase in the number of medical interventions, and the necessity to constantly maintain stricter safety precautions. According to the literature, predictive factors for work-related stress also include demographic factors [5,6]. Studies of health care workers performing their duties during the COVID-19 pandemic have shown a higher risk of depression, anxiety and psychological disorders, and the severity of symptoms correlates directly with demographic factors such as age, gender, and occupation [7]. Emergency medical systems around Europe differ greatly in terms of organisation, forms of employment and the competencies of personnel, however, a common feature for all is that during the pandemic emergency systems are particularly at risk from coronavirus infection. This paper attempts to assess the effect of demographic factors such as age, gender, profession, and place of work on the level of work-related stress experienced during the COVID-19 pandemic in seven European countries.

## 2. Materials and Methods

The research was conducted during the COVID-19 pandemic between the 27 March 2020 and the 20 April 2020. The main research hypothesis was that among emergency medical personnel, age, workplace, profession and gender affects the level of professional stress experienced while carrying out work-related duties during the COVID-19 pandemic. The study was carried out in such a way as to ensure the anonymity of the respondents. An internet-based questionnaire format was adopted for the study. The research tool was made available to respondents via social media and information on the websites of institutions involved in the study.

The study project obtained a positive assessment from The Ethics Committee of the University of Bielsko-Biała (Decisions no. 2020/03/1/1).

• Study group

The research included 1984 employees of emergency medical systems in seven countries—the Czech Republic, Finland, Spain, Norway, Poland, Slovakia, and the United Kingdom. Emergency medical systems in European countries differ in terms of organisation, forms of employment and the competencies of personnel. In all the countries included in the study, the emergency medical system comprises Medical Response Teams and Hospital Emergency Wards. During the study, the level of virus spread varied between countries. In each of the countries surveyed, personal protective equipment for workers was introduced and changes were made in the operation of emergency systems. The survey was addressed to all employees of rescue systems in each country. It is difficult to determine the exact number of people working in each system, as there are very large differences in employment, organization of rescue systems and public reporting of these data. This fact may be a limitation of the survey. The study group included doctors, paramedics, and nurses. All persons in the study group are medical professionals working with emergency patients, and it should be assumed that any such contact constitutes an exposure to infection, which is also related to the performance of professional medical procedures in direct contact with patients and exposure to aerosols in situations such as endotracheal intubation.

• Inclusion and exclusion criteria

Criteria for inclusion in the research were achieving a representative number of respondents and providing answers to all the questions in the questionnaire. Initial analysis excluded Sweden and Denmark from the study as only five questionnaires were received from each country. For two of the study questions (Table 1 and Table 2), results for respondents from Slovakia were not used as they were all paramedics working only in Medical Response Teams. For one of the study questions (Table 2), statistical analysis of the results for respondents from the Czech Republic, Finland and Spain, was only conducted on two professional groups. Table 1 presents the statistical correlation in each country between the stress experienced and study participants’ place of work in the emergency medical system. No statistically significant differences were found in stress levels according to place of work.

Table 2 presents statistical analysis of experienced work-related stress according to profession. It can be observed that individual study groups differ from each other, with the highest level of stress experienced by nurses from Spain and Poland, while in the remaining groups no statistically significant differences were observed.

The demographic characteristics of the study group are presented in Table 3.

• Research methods and tools

The research tool was a proprietary internet questionnaire. Before the study was begun, the questionnaire was validated using the Ω Mc-Donald test, achieving a result of >0.7, indicating a satisfactory level of reliability [8]. The questionnaire consisted of 24 questions and was divided into three sections. The first section contained information on the aim of the research, a voluntary consent clause for participation in the study and information on the use of the results in a subsequent research paper. The second section consisted of questions regarding study participants’ demographic characteristics, such as: age, gender, profession, place of work in the emergency medical system, and work experience. The third section related to the level of work-related stress experienced while carrying out emergency medical procedures in pandemic conditions, and respondents’ opinion on the functioning of the healthcare system and working conditions during the pandemic. In order to verify the assumed research hypothesis, analysis was conducted of respondents’ answers to the question “What levels of work-related stress do you experience while conducting your professional duties during the COVID-19 pandemic?” To objectify the answers provided, a five-point Likert scale was used to determine the degree of a given phenomenon, from none (1—I do not experience stress) to a high level (5—I experience a high degree of stress). The workload of medical professionals during the pandemic was very high, so the authors adopted a Likert scale survey model. This type of survey was made possible by the use of mobile devices and the questions contained in the questionnaire were formulated in a simple, concise manner so as to allow the respondents to answer in the shortest possible time. For each country included in the study, statistical correlation was conducted between the levels of stress experienced by respondents and the designated demographic factors. In order to obtain reliable answers, the questionnaire was translated into each language using the committee translation method. Each version of the questionnaire was translated into the relevant language by two independent translators, and for validation was later translated back into the original language so as to minimise the risk of respondents not understanding individual questions [9].

• Statistical analysis

The level of significance adopted for the statistical analysis was *p* = 0.05. Non-parametric U Mann–Whitney or Kruskal–Wallis tests were used to analyse the presented quantitative variables divided by group. In order to determine which differences between the studied groups were significant, comparison in pairs was applied using the post hoc Bonferroni test. The tests were selected based on the distribution of variables, which was verified using the Shapiro–Wilk test. Calculations were carried out in the R statistical environment, version 3.6.0, PSPP software, and MS Office 2019.

## 3. Results

The results presented in Table 4 shows the increase in cases of COVID-19 from the start to the end of the survey in each European country.

The results presented in Table 5 show the differences in work-related stress experienced by emergency medical system personnel according to country. The statistical analysis applied showed statistically significant differences (*p* < 0.001). The highest level of stress was experienced by personnel in the United Kingdom M = 4.03, and the lowest by Norwegian employees M = 2.89.

Table 6 presents experienced work-related stress in individual countries according to gender. In the statistical majority, differences were obtained indicating that women actively working in the healthcare system during the pandemic experienced higher stress levels than men.

Table 7 presents experienced work-related stress during the COVID-19 pandemic according to age. No statistically significant differences were found between different age groups during the COVID-19 pandemic.

## 4. Discussion

Stress is a part of medical professions irrespective of in what form and what place of work duties are carried out. Research on the topic clearly indicates that medical professions are subject to particularly high levels of stress due to many aspects of work duties. It is very difficult to assess the level of stress among such a diverse study group as many factors come into play. Our research has shown statistically significant differences in the level of experienced stress within the study group populations. Such research is pioneering and has not previously been conducted, which is why referring to results from the literature is impossible. During our considerations, we correlated the level of stress experienced with the increase in contracting illnesses during the study. The highest levels of stress were indicated by the British (ME 4.03), with a more than eightfold increase in illness, while the lowest level was amongst Norwegians (ME 2.98), with a close to twofold increase in illness. It can therefore be assumed that the spread of the pandemic has had an effect on the level of work-related stress experienced by emergency medical personnel. Kaburi [10] demonstrates that working with patients generates high and extreme levels of stress in all personnel. In their work, Halpern [3] shows that stress levels among ambulance workers are particularly highlighted and documented. Johnson [4] proved that one of the professions most severely affected by stress are medical response team personnel and shows in the same study that medics working in hospitals are better able to cope with stress levels. Our research compares the level of experienced stress between emergency system personnel working on hospital emergency wards and medical response teams. Differences in the level of experienced stress were observed only in the Spanish emergency medical system. Ambulance workers had a significantly higher level of stress than emergency department personnel. Many factors may affect this, such as the preparedness of the system, appropriate protection, and the degree of the spread of the pandemic in the study period. Research into the level of experienced stress according to gender are inconclusive and the presented results display differences from one another. Vermulen et al. [11] clearly indicate that professional workload generates stress among both women and men. Povedano Himenez [12] created a matrix for the predictors of work-related stress using the example of Spanish healthcare personnel and showed that men display a greater resistance to stress than women. A study by Emily o’Dowd [13] demonstrated than women working in the health service are more susceptible to stress than men. These results were confirmed in a paper by Wu [14]. Our research confirms that women in the study group suffered stress decidedly more often in their professional life. In most of the study population, statistically significant differences were found in the level of stress experienced by women and men. Women employed in the emergency systems in Finland, Slovakia, Poland, and Norway experienced greater stress than men. Discussion on the conditions for the incidence of stress among women and men in European countries is difficult. To date, there is no similar research in the literature that compares the incidence of stress among medical personnel according to country. Many factors may affect the level of stress experienced, such as workload directly related to the pandemic or the functioning of the emergency medical system. In their research, Segerstrom and Miller [15] prove that age affects the ability to cope with stressful situations. In their deliberations on the issue of the effect of age on the level of experienced stress, the researchers indicated a dependency related to the functioning of the hormonal and immune systems in experiencing stress, which emphasised that in old age stress was experienced more acutely [16]. An additional element that may generate higher perceived stress is that older people are more likely to be infected with the virus [17]. Our research did not show any statistically significant differences in any of the countries included in the study with regard to the level of stress experienced according to the age of the personnel studied.

## 5. Conclusions

The spread of the COVID-19 pandemic has increased the level of work-related stress experienced in the studied European populations. Women working in European emergency medical systems are more vulnerable to work-related stress while carrying out emergency medical procedures during the pandemic. Age does not have a statistically significant effect on the incidence of work-related stress, and differences in the level of stress experienced while carrying out duties in pre-hospital conditions were only found among Spanish emergency medical system personnel. Analysing the results obtained; it can be concluded that practical solutions of psychological assistance should be introduced to reduce work-related stress in the group of emergency system personnel with a special focus on pre-hospital system workers.

## Figures and Tables

**Table 1:** Level of experienced work-related stress depending on place of work by country.

				Descriptive Statistics				
Name	Place of Work	U	*p*	M	SD	Min	Max	Me
Czech Republic	Hospital/A&E	1548.50	0.802	3.49	1.10	1.00	5.00	4.00
	Response Teams			3.56	0.88	2.00	5.00	4.00
Finland	Hospital/A&E	419.50	0.559	3.50	0.76	3.00	5.00	3.00
	Response Teams			3.24	1.08	1.00	5.00	3.00
Spain	Hospital/A&E	1145.50	0.007	3.97	0.92	2.00	5.00	4.00
	Response Teams			3.54	0.58	2.00	4.00	4.00
Norway	Hospital/A&E	2108.00	0.162	3.31	1.08	1.00	5.00	3.00
	Response Teams			2.96	1.06	1.00	5.00	3.00
Poland	Hospital/A&E	106,261.00	0.332	3.76	1.11	1.00	5.00	4.00
	Response Teams			3.69	1.10	1.00	5.00	4.00
United Kingdom	Hospital/A&E	1477.00	0.181	3.83	0.97	2.00	5.00	4.00
	Response Teams			4.08	0.86	1.00	5.00	4.00

U—test statistic; *p*—statistical significance; M—mean; SD—standard deviation; Me—median; Min—minimum result; Max—maximum result, A&E—accident & emergency department.

**Table 2:** Level of stress according to profession by country.

						Descriptive Statistics				
Name	Profession	Χ^2^	df	U	*p*	M	SD	Min	Max	Me
Czech Republic	Nurse			1517.50	0.951	3.51	1.11	1.00	5.00	4.00
	Paramedic					3.55	0.90	2.00	5.00	4.00
Finland	Nurse			1873.00	0.587	3.31	1.07	1.00	5.00	3.00
	Paramedic					3.22	1.06	1.00	5.00	3.00
Spain	Doctor					3.35	1.06	2.00	5.00	3.00
	Nurse			760.50	0.014	3.96	0.84	2.00	5.00	4.00
Norway	Doctor					2.67	0.58	2.00	3.00	3.00
	Nurse	0.26	2		0.879	2.96	1.01	1.00	5.00	3.00
	Paramedic					2.99	1.09	1.00	5.00	3.00
Poland	Doctor					3.73	1.11	1.00	5.00	4.00
	Nurse	12.12	2		0.002	3.92	1.14	1.00	5.00	4.00
	Paramedic					3.66	1.09	1.00	5.00	4.00
United Kingdom	Doctor					3.79	0.98	2.00	5.00	4.00
	Nurse	141	2		0.495	3.92	1.00	2.00	5.00	4.00
	Paramedic					4.07	0.87	1.00	5.00	4.00

U—test statistic; *p*—statistical significance; M—mean; SD—standard deviation; Me—median; Min—minimum result; Max—maximum result.

**Table 3:** Statistical analysis of study group.

Country	Frequency	Percentage
Czech Republic	117	5.90%
Finland	127	6.40%
Spain	155	7.80%
Norway	345	17.40%
Poland	955	48.10%
Slovakia	136	6.90%
United Kingdom	149	7.50%
Profession	Frequency	Percentage
Doctor	160	8.10%
Nurse	549	27.70%
Paramedic	1275	64.30%
Place of work	Frequency	Percentage
Hospital Emergency Department	646	32.60%
Response Teams	1338	67.40%
Age	Frequency	Percentage
18–30	701	35.30%
31–40	741	37.30%
41–50	390	19.70%
51–60	135	6.80%
over 60	17	0.90%
Gender	Frequency	Percentage
Female	786	39.60%
Male	1198	60.40%
Work experience	Frequency	Percentage
up to 5 years	605	30.50%
6–15 years	857	43.20%
16–30 years	449	22.60%
over 30 years	73	3.70%

**Table 4:** Increase in cases of COVID-19 during the study period by country.

Country	Number of Cases Diagnosed on the Date of Beginning of Survey	Number of Cases Diagnosed on the Day of the End of the Survey	Multiplication Rate
U.K.	13,182	113,015	8.57
Poland	1389	9593	6.90
Slovakia	292	1172	4.013
Finland	1041	3868	3.71
Czech Republic	2027	6758	3.333
Norway	3771	7156	1.897
Spain	150,518	227,936	1.514

**Table 5:** Differences in work-related stress experienced by emergency system personnel in the countries studied.

				Descriptive Statistics				
Country	Χ^2^	df	*p*	M	SD	Min	Max	Me
	184.96	6	<0.001					
Czech Republic				3.51	1.02	1.00	5.00	4.00
Finland				3.26	1.06	1.00	5.00	3.00
Spain				3.90	0.88	2.00	5.00	4.00
Norway				2.98	1.06	1.00	5.00	3.00
Poland				3.72	1.10	1.00	5.00	4.00
Slovakia				3.16	1.15	1.00	5.00	3.00
United Kingdom				4.03	0.88	1.00	5.00	4.00

Χ^2^—test statistic; df—degrees of freedom; *p*—statistical significance; M—mean; SD—standard deviation; Min—minimum result; Max—maximum result; Me—median.

**Table 6:** Differences in experienced work-related stress depending on gender.

				Descriptive Statistics				
Name	Gender	U	*p*	M	SD	Min	Max	Me
Czech Republic	Female	1211.00	0.210	3.58	1.03	1.00	5.00	4.00
	Male			3.35	1.01	2.00	5.00	3.00
Finland	Female	1486.00	0.009	3.54	0.99	1.00	5.00	3.00
	Male			3.01	1.07	1.00	5.00	3.00
Spain	Female	1669.00	0.228	3.94	0.86	2.00	5.00	4.00
	Male			3.74	0.96	2.00	5.00	4.00
Norway	Female	10,260.50	0.014	3.19	1.05	1.00	5.00	3.00
	Male			2.89	1.06	1.00	5.00	3.00
Poland	Female	74,833.50	<0.001	4.05	1.00	1.00	5.00	4.00
	Male			3.56	1.12	1.00	5.00	4.00
United Kingdom	Female	2555.50	0.682	4.08	0.82	2.00	5.00	4.00
	Male			3.99	0.93	1.00	5.00	4.00
Slovakia	Female	1565.00	0.004	3.52	1.16	1.00	5.00	3.00
	Male			2.94	1.09	1.00	5.00	3.00

U—test statistic; *p*—statistical significance; M—mean; SD—standard deviation; Me—median; Min—minimum result; Max—maximum result.

**Table 7:** Experienced work-related stress according to age.

					Descriptive Statistics				
Name	Age	Χ^2^	df	*p*	M	SD	Min	Max	Me
Czech Republic	18–30	1.53	2	0.465	3.48	0.97	1.00	5.00	4.00
	31–40				3.41	0.89	2.00	5.00	3.00
	over 40				3.63	1.17	1.00	5.00	4.00
Finland	18–30	0.23	2	0.889	3.25	1.08	1.00	5.00	3.00
	31–40				3.30	1.12	1.00	5.00	3.00
	over 40				3.18	0.91	1.00	5.00	3.00
Spain	18–30	1.14	2	0.567	3.81	0.95	2.00	5.00	4.00
	31–40				4.03	0.83	2.00	5.00	4.00
	over 40				3.89	0.86	2.00	5.00	4.00
Norway	18–30	1.68	2	0.431	2.89	1.02	1.00	5.00	3.00
	31–40				2.97	1.09	1.00	5.00	3.00
	over 40				3.05	1.07	1.00	5.00	3.00
Poland	18–30	0.65	2	0.724	3.70	1.04	1.00	5.00	4.00
	31–40				3.75	1.10	1.00	5.00	4.00
	over 40				3.69	1.22	1.00	5.00	4.00
United Kingdom	18–30	0.95	2	0.621	3.98	0.77	2.00	5.00	4.00
	31–40				4.10	0.90	1.00	5.00	4.00
	over 40				4.00	0.99	2.00	5.00	4.00
Slovakia	18–30	1.81	2	0.404	3.04	1.09	1.00	5.00	3.00
	31–40				3.11	1.18	1.00	5.00	3.00
	over 40				3.36	1.19	1.00	5.00	3.00

Χ^2^—test statistic; df—degrees of freedom; *p*—statistical significance; M—mean; SD—standard deviation; Min—minimum result; Max—maximum result; Me—median.

## Data Availability

The data presented in this study are available on request from the corresponding author.
